# Overview of dual console robotic training for surgical residents: a systematic review

**DOI:** 10.1007/s11701-026-03331-7

**Published:** 2026-04-10

**Authors:** Rhieya Rahul, Pratyush Kumar, Matthew G. Davey, Noel E. Donlon, Christina Fleming, Jarlath C. Bolger

**Affiliations:** 1 School of Medicine, Royal College of Surgeons in Ireland, Dublin, D02 YN77 Ireland; 2 Department of Surgery, Royal College of Surgeons in Ireland, Dublin, D02 YN77 Ireland; 3 Department of Colorectal Surgery, University Hospital Limerick, Dooradoyle, Limerick, V94 F858 Ireland; 4 School of Medicine, University of Limerick, Limerick, Ireland; 5 Division of Upper GI Surgery, Beaumont Hospital, Dublin, D09 V2N0 Ireland

**Keywords:** Robotic surgery, Dual console system, Single console system, Da Vinci robotic system, Da Vinci surgical system, Robotic assisted training

## Abstract

Dual-console robotic systems represent a paradigm shift in surgical education, allowing real-time shared control and instructor intervention. Although increasingly adopted, their impact across specialties, educational effectiveness, and implementation barriers are not fully established. To perform a systematic review to provide a comprehensive overview of dual – console training in robotic surgery by synthesizing available evidence regarding training outcomes, implementation strategies, educational effectiveness, and the broader impact of dual – console systems on surgical education and surgical performance. A systematic search was performed as per PRISMA guidelines. Descriptive statistics were recorded and analysed using SPSS v26.0. Overall, 12 studies including 1,267 participants were included. These included residents from gynecology, urology, colorectal surgery, general surgery, and hepatopancreatobiliary surgery. Dual consoles consistently increased trainee operative exposure, autonomy scores, and skill acquisition, with improvements in suturing times and procedural step completion. Safety outcomes were preserved or improved, however operative efficiency varied with some studies demonstrating reduced operative times, while others reported neutral or slightly prolonged procedures due to greater trainee participation. Trainee and instructor satisfaction was uniformly high, citing improved communication, confidence, and educational quality. Implementation barriers included cost, curriculum integration, and faculty development needs. Dual-console systems significantly enhance robotic surgical training by improving technical skill acquisition, trainee engagement, and perceived educational quality, while maintaining patient safety. Operative efficiency outcomes are variable and context-dependent. Despite clear educational advantages, widespread adoption is limited by financial and curricular barriers. Future efforts should focus upon the integration of dual-console training into surgical training curricula.

**Supplementary Information:**

The online version contains supplementary material available at 10.1007/s11701-026-03331-7.

## Introduction

The evolution of robotic-assisted surgery has brought unprecedented precision and minimally invasive capabilities to surgical practice, necessitating specialised training methodologies to ensure competent robotic surgeons [1]. More recent robotic platforms have seen the development of dual-console systems, which have created new opportunities for surgical training by enabling simultaneous operating and instruction by trainers at the robotic console [2].

The dual-console system represents a novel training strategy which builds on the traditional apprenticeship model, the foundation of surgical training for decades. Similarly, the dual-console system facilitates initial observation at the console with the trainer, followed by graduated autonomy where the trainer has the ability to reassume the primary operator role at any stage during the procedure [3]. This novel technology allows trainees to engage directly with robotic controls while receiving real-time guidance from their trainer at a parallel console, creating unique trainee-trainer dynamics which are unachievable at single-console robots or even in laparoscopic surgery [3]. These systems facilitate immediate feedback, allow for seamless transitions between instructor and trainee control if necessary, and provide opportunities for collaborative problem-solving during complex surgical scenarios.

The implementation of dual-console training has shown promising results across various surgical specialties, with early reports suggesting educational advantages, including greater trainee involvement in procedural steps and opportunities for more interactive teaching [2, 4]. At the same time, barriers such as equipment cost, limited availability, and the need for curriculum adjustments have been noted across institutions [5].

Despite increasing interest, current literature on dual-console training spans multiple surgical specialties and study designs, with variable outcome measures and heterogeneous findings. This variability makes it difficult for educators and program directors to draw definitive conclusions about the overall effectiveness and best practices for implementation.

The purpose of this review is therefore to systematically evaluate existing evidence on dual-console training, synthesizing reported benefits, limitations, and implementation considerations in order to guide future directions in robotic surgical education.

The aim of this study was to perform a systematic review to provide a comprehensive overview of dual-console training in robotic surgery by synthesising available evidence regarding training outcomes, implementation strategies, educational effectiveness, and the broader impact of dual-console systems on surgical education and surgical performance.

## Methods

### Search strategy

A systematic literature search was conducted on June 10, 2025, across three major electronic databases: PubMed, Scopus, and EMBASE in accordance with the Preferred Reporting outcomes for Systematic Review and Meta-Analysis (PRISMA) guidelines [6]. The search strategy was tailored to each database to optimize retrieval of relevant studies, with the search terms that yielded the most results being selected for each respective database.

*Search Terms Applied*:

1. *Scopus Search Strategy* **-** (“robotic surgical training” OR “robot-assisted training” OR “dual console robotic”) AND (“residents” OR “trainees” OR “fellows” OR “medical education”).
2. *Pubmed and EMBASE Search Strategy* **-** (‘dual console’ OR ‘dual-console’ OR ‘robotic surgery system’/exp OR ‘robotic surgery system’ OR ‘robot-assisted surgery’/exp OR ‘robot-assisted surgery’ OR ‘robotic-assisted surgery’) AND (‘surgical training’/exp OR ‘surgical training’ OR ‘surgical education’/exp OR ‘surgical education’ OR ‘resident training’/exp OR ‘resident training’ OR ‘fellow training’ OR ‘skill acquisition’/exp OR ‘skill acquisition’ OR ‘simulation training’/exp OR ‘simulation training’) AND (‘trainee’/exp OR ‘trainee’ OR ‘resident’/exp OR ‘resident’ OR ‘fellow’/exp OR ‘fellow’ OR ‘junior surgeon’ OR ‘novice surgeon’).

The search was conducted using the “All Fields” option for each database to maximize sensitivity and ensure comprehensive retrieval of relevant literature. The search strategy was intentionally tailored to the indexing conventions of each database; Scopus does not support MeSH or Emtree controlled vocabulary and therefore required a free – text approach, whereas Pubmed and EMBASE support exploded subject headings. Despite these structural differences, all strategies were designed to capture the same conceptual domains.

### PICOTT framework

The research question was structured using the PICOTT (Population, Intervention, Comparison, Outcomes, Timing, Type of Study) framework to ensure systematic and comprehensive evaluation:

### Inclusion and exclusion criteria

**Inclusion Criteria**

- Peer-reviewed studies published in English.
- Studies examining the impact of dual-console systems in robotic-assisted surgery training.
- Participants include surgical trainees (i.e.: residents or fellows).
- Quantitative or qualitative evaluation of training outcomes.
- Studies published between 2010 and 2025.
- Randomised controlled trials (RCTs), including but not limited to: RCT’s, prospective and retrospective cohort studies, case series, cross – sectional educational research, qualitative and mixed – methods studies, feasability and pilot studies and any other studies with original primary data.

**Exclusion Criteria**

- Reviews, editorials, or opinion pieces without original data.
- Non-English language studies.
- Studies that did not explicitly examine dual-console systems.
- Studies without explicit training comparisons.
- Non-peer-reviewed opinions or pre-2010 publications.
- Studies focused solely on technical implementation without trainee outcomes.
- Studied with results for less than 5 participants / patients.
- Studies not meeting the aforementioned inclusion criteria.

### Study selection and screening

Following searches of the electronic databases, all retrieved citations were imported into Covidence systematic review software for screening and data management. Two independent reviewers conducted the screening process for each study to minimize selection bias and ensure comprehensive evaluation.

The screening process consisted of two phases:

1. *Title and Abstract Screening*: Two reviewers independently screened all titles and abstracts against the predefined inclusion and exclusion criteria.
2. *Full-Text Screening*: Studies that met initial screening criteria underwent full-text review by two independent reviewers.

Discordance of opinions between reviewers at any stage of the screening process were resolved through discussion, and when necessary, a third reviewer was consulted to reach consensus.

### Data extraction

Data extraction was performed independently by two reviewers using a standardized data extraction form developed specifically for this review. The extracted data encompassed comprehensive study characteristics including: (1) author(s), (2) year of publication (3) level of evidence (4) study design (5) surgery assessed (6) country (7) total participants/patients (8) single console number (9) dual console number (10) outcome measures (11) complications (12) reported operative duration/times (13) skill level measured and (14) other outcomes. Participant information was systematically recorded, documenting total participants or patients involved in each study, with specific enumeration of single console and dual console group sizes. Outcome measures were comprehensively catalogued, along with any reported complications and operative duration or procedural times. The skill level measurement methodologies employed in each study were documented, as were any additional outcomes reported by the investigators. This systematic approach to data extraction ensured consistent capture of all relevant information necessary for comprehensive analysis and synthesis of the available evidence.

### Outcome measures

#### Comparative analysis

Where studies included both a single-console and a dual-console group, a direct comparison of outcomes between modalities was pre-specified as a secondary analysis. Outcomes compared included operative time, complication rates, trainee autonomy scores, operative exposure (console time, surgical steps performed), and trainee satisfaction. Given the heterogeneity of study designs and outcome reporting, a narrative synthesis was employed; pooled quantitative analysis was not performed. Studies reporting only single-arm dual-console data without a comparator group were included for descriptive synthesis of training outcomes, implementation strategies, and educational effectiveness.

**Primary outcomes**

- Training Outcomes - technical skill acquisition (structured autonomy scores, knot-tying and suturing times, operative steps completed, simulation task performance), trainee autonomy progression (e.g., graduated autonomy scores, proportion of cases with high trainee involvement), and operative exposure (console time, console-to-docking ratio, surgical steps performed by trainee) [7–11].
- Educational Effectiveness - trainee and instructor satisfaction scores, perceived educational quality, communication during training, and qualitative indicators such as situational awareness and time to proficiency in defined surgical tasks [12–14].

**Secondary outcomes**

- Implementation strategies – reported approaches to integrating dual-console systems into training curricula, including modular step-by-step frameworks, faculty development programmes, and case allocation methods.
- Instructor feedback and assessment scores.
- Trainee confidence and satisfaction.
- Long-term skill retention and transfer.
- Implementation barriers – reported challenges to adoption of dual-console systems including financial costs, equipment availability, curricular integration difficulties, faculty development requirements, and scheduling constraints.

### Risk of bias assessment

Risk of bias assessment was conducted independently by two reviewers for all included studies. The assessment tools used were tailored to the study designs encountered we used the Newcastle Ottawa scale [15] for almost all studies as well as a more specialised tool based on the specific studies we included, these tools were:

- RCTs: Cochrane Risk of Bias 2 (RoB 2) tool [16].
- Non-randomized studies: Risk of Bias in Non-randomized Studies of Interventions (ROBINS-I) tool [17].
- Qualitative studies: Critical Appraisal Skills Programme (CASP) qualitative research checklist [18].

### Quality assessment

The overall quality of evidence was assessed using the Grading of Recommendations Assessment, Development and Evaluation (GRADE) approach [19] for each outcome of interest. This assessment considered study design, risk of bias, inconsistency, indirectness, imprecision, and other factors that might affect the confidence in effect estimates.

## Results

### Study selection

**Fig. 1:**
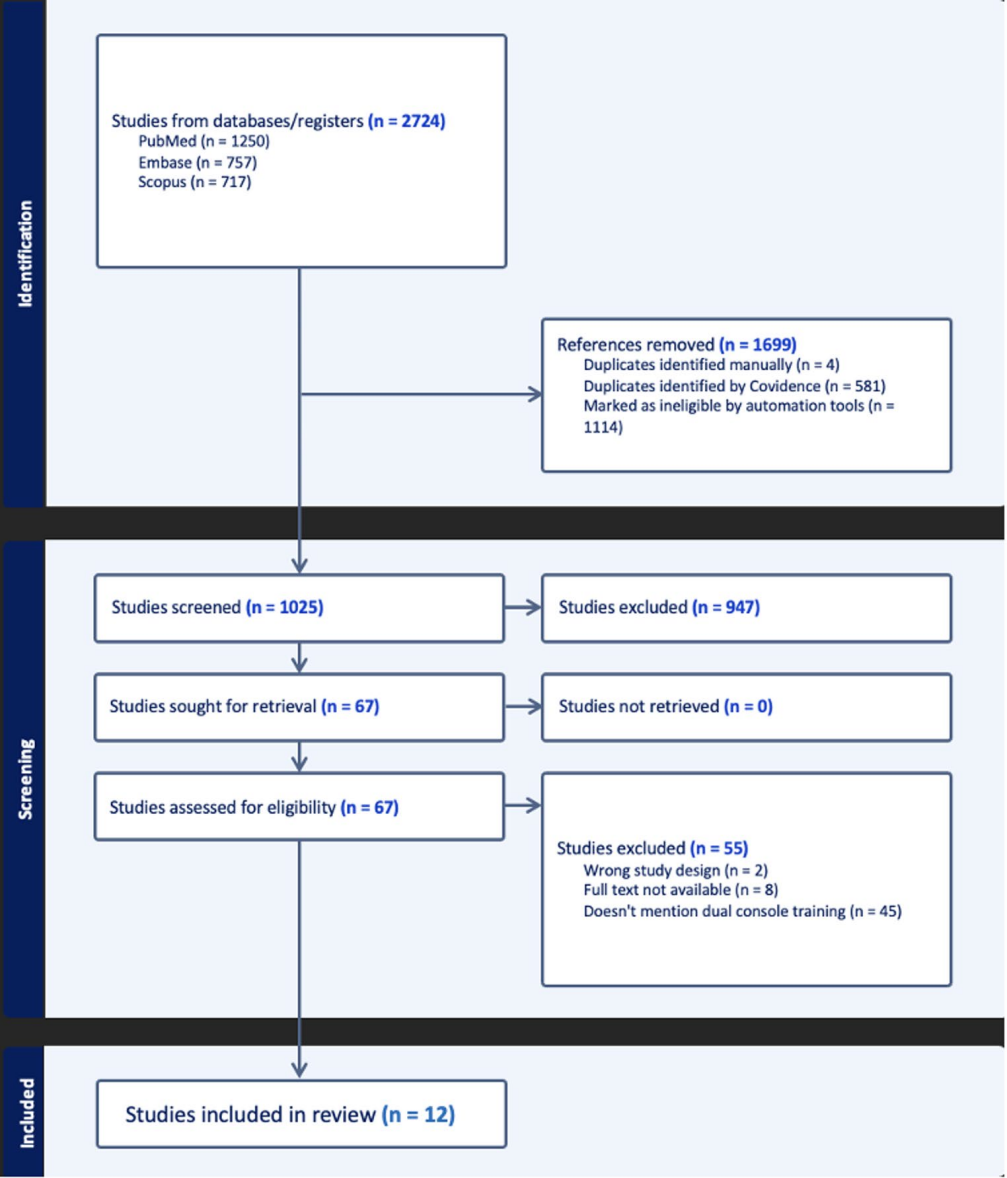
PRISMA flow chart

The electronic systematic search across PubMed, Scopus, and EMBASE databases yielded a total of 2724 records before deduplication. After removing duplicates, 1025 unique records remained for screening. Of these, 947 records were excluded and the remaining 68 studies underwent full-text assessment for eligibility. Ultimately, 12 studies met the inclusion criteria and were included in the final qualitative synthesis. Figure 1 demonstrates the PRISMA flowchart outlining the systematic search process.

### Study characteristics

The 12 included studies were published between 2012 and 2025, representing a diverse range of surgical specialties and geographic locations. Studies originated from the United States (58.3%, 7/12), France (*n* = 16.7%, 2/12), Italy (*n* = 8.3%, 1/12), Switzerland (*n* = 8.3%, 1/12), and Japan (*n* = 8.3%, 1/12), with one study conducted in Australia.

The study designs varied considerably, including one RCT, four prospective cohort studies, three retrospective cohort studies, two case series, one qualitative observational study, and one experimental animal study. Surgical specialties represented included gynecology and gynecologic oncology (*n* = 50%, 6/12), urology (*n* = 16.6%, 2/12), general surgery and colorectal surgery (*n* = 16.6%, 2/12), hepatopancreatobiliary surgery (*n* = 8.3%, 1/12), and general robotic surgery training (*n* = 8.3%, 1/12). A total of 1,267 participants were included across all studies, ranging from 7 to 381 participants per study. Training levels encompassed medical students (*n* = 8.3%, 1/12), residents across various postgraduate years (*n* = 66.6%, 8/12 studies), and fellows (*n* = 50%, 6/12 studies), with some studies including multiple training levels. Table 1 outlined the study characteristics.

**Table 1:** Study characteristics

S No	Author(s)	Year	Level of evidence	Study design	Surgery assessed	Country
1.	Crusco et al. [10]	2014	Level I (Randomized Controlled Trial)	Randomized block RCT in a training lab	Robotic suturing/knot tying tasks (simulation)	USA
2.	Jackson et al. [12]	2020	Expert opinion/Technical report (Level V)	Descriptive educational ‘How I Do It’ paradigm	HPB & Advanced GI robotic cases (hiatal hernia, distal pancreatectomy, pancreaticoduodenectomy)	USA
3.	Cristofari et al. [13]	2022	Qualitative observational study	Video-based qualitative analysis of dual-console training	Robotic Roux-en-Y gastric bypass	Switzerland
4.	Landry et al. [22]	2022	Level III (Retrospective cohort study)	Retrospective comparison of single vs. dual console in colorectal surgery with residents	Various colorectal surgeries (majority colon resections)	USA
5.	Klapczynski et al^7^.	2021	Pilot prospective observational study	Single-centre feasibility study of resident-performed robotic hysterectomy using dual console	Robot-assisted hysterectomy for endometrial cancer or adenomyosis	France
6.	Marengo et al. [23]	2012	Level II (Prospective cohort study)	Prospective cohort in teaching hospital	Various gynecologic robotic-assisted laparoscopic procedures	Italy
7.	Leon et al. [9]	2022	Level II (Prospective observational study)	Prospective comparative analysis (single vs. dual console)	Robotic hysterectomy	USA
8.	Margueritte et al. [8]	2020	Level IV (Case Series)	Prospective, single-center observational study	Robot-assisted gynecological surgery (hysterectomy, myomectomy, sacrocolpopexy)	France
9.	Morgan et al. [20]	2015	Level III (Retrospective Cohort)	Retrospective, single-center observational study	Robot-assisted laparoscopic prostatectomy (RALP)	USA
10.	Williams et al. [11]	2025	Level III (Prospective Cohort)	Prospective cohort study	Robot-assisted laparoscopic prostatectomy (RALP)	Australia
11.	Takahashi et al. [14]	2024	Level IV (Case Series/Experimental Study)	Experimental animal study with simulated telesurgery conditions	Gastrectomy, rectal resection, cholecystectomy, and bleeding control tasks (robotic surgery)	Japan
12.	Smith et al. [21]	2012	Level III (Retrospective Comparative Study)	Retrospective cohort study	Laparoscopic vs. robotic gynecologic oncology procedures (hysterectomy, lymphadenectomy)	USA

### Training outcomes

#### Technical skill and trainee autonomy

Overall, five studies reported outcomes in relation to technical performance (41.7%). Technical skill assessment methodologies varied considerably across the included studies. Klapczynski et al. employed a comprehensive 21-step autonomy score for resident-performed robotic hysterectomy, reporting a mean score of 29.8 out of 42 possible points. The study demonstrated significant improvement in knot-tying times from 335 s to 270 s (*p* = 0.043) over the course of dual console training [7].

Margueritte et al. similarly reported improvement in suturing times between first and second procedures, with mean times decreasing from 3 min 38 s to 3 min 14 s (*p* = 0.033) [8]. Leon et al. outlined that dual console training resulted in minimally invasive gynecological surgery (MIGS) fellows performing significantly more surgical steps compared to single console training (Odds ratio 3.37), with increased hands-on training opportunities without compromising patient safety [9].

The simulation-based study by Crusco et al. evaluated task performance in novice medical students using standardised robotic suturing tasks. While no significant differences were observed in task completion times between single and dual console groups across four different knot-tying tasks, the dual console system demonstrated advantages in providing “virtual hands” guidance and facilitating easier control swapping between instructor and trainee [10].

Williams et al. implemented a modular 13-step training approach for robot-assisted laparoscopic prostatectomy, with trainees completing more than 75% of operative steps in 31% of cases, demonstrating progressive skill acquisition through dual console mentorship [11] (Table 2).

**Table 2:** Results database

S No	Author(s)	Total participants/patients	Single console (*n*)	Dual console (*n*)	Outcome measures	Complications reported	Operative duration/times	Skill level measured	Other outcomes
1.	Crusco et al. [10]	40	20	20	Task times for 4 knots; frayed sutures; floating knots	Not applicable (simulation study)	Interrupted 2 H: 300s vs. 294s; Interrupted 1 H: 198s vs. 212s; Fig-8 2 H: 261s vs. 219s; Fig-8 1 H: 200s vs. 199s (no significant differences)	Novice medical students (no residents/fellows)	Dual console allowed for “virtual hands” guidance and easier control swapping. Potential advantages in safety and trainer intervention speed (not quantitatively measured)
2.	Jackson et al. [12]	Not specified (15 trainees progressed over 4 years)	N/A	All training on dual console	Time/number of cases to achieve tasks (e.g., advanced hiatal dissection within ~ 5 cases)	Not reported	Not reported	Residents & fellows (PGY3+)	Higher trainee satisfaction due to maximized console time (no bedside-only phase) Parallel learning for faculty and trainee (faculty maintains skill while teaching). Increased team interactivity vs. traditional robotic console teaching
3.	Cristofari et al. [13]	10 procedures observed; 3 sequences analyzed in depth	None (dual-console context)	All observed training with dual console	Trainee communication, three-hand fluency, situational awareness; didactic use of pointers and control handover	Not the focus/not detailed	Not reported (qualitative focus)	Surgeon in training (residents/fellows)	Identified tension between quiet, efficient OR environment and need for verbalization in training Suggested use of video-based self-confrontation debriefing to enhance reflection and skill acquisition. Highlighted mental load on lead surgeon when supervising both trainee at second console and novice bedside assistant
4.	Landry et al. [22]	131	71	60	Operative time, conversion to open, ICU admission, length of stay, transfusion, surgical margin, lymph nodes harvested, anastomotic leak, readmission, wound infection, reoperation	No significant difference between single and dual console in complications: ICU admission, anastomotic leak, wound infection, reoperation	Single: 220.0 ¬± 74.5 min; Dual: 200.6 ¬± 61.5 min (*p* = 0.111, not significant)	No direct skill assessment of residents; focus on patient outcomes	Non-significant decrease in operative time (dual: 200.6 min vs. single: 220.2 min); trend toward more lymph nodes harvested in dual console group
5.	Klapczynski et al. 7	7	0	7	Conversion to laparotomy, complications, operative time, suturing time, 21-step autonomy score, NASA-TLX workload	No conversions, no intra- or post-operative complications	Mean total op time: 166 ¬± 12 min; Resident-performed portion (21 steps): 104 ¬± 23 min; Knot times improved from 335s to 270s	21-step autonomy score (mean 29.8/42); knot-tying speed improvement; identification of difficult steps (ureter identification, suturing); older residents scored higher	Mean autonomy score 29.8/42; improvement in knot tying time from 335s to 270s (*p* = 0.043); most difficult steps were ureter identification & knot tying; older residents scored higher
6.	Marengo et al. [23]	33	0	33	Docking time, operative time, anesthesia time, hemoglobin drop, conversion rate, hospital stay	4 (12%): transient ischemic attack, port-site hernia, periumbilical hematoma, vaginal cuff hematoma	Mean docking 22 min; mean operative 152 min; mean anesthesia 196 min	Experienced laparoscopic surgeons with resident assistance	Double console allowed mentoring; faster adaptation likely due to prior lap experience; stepwise start with simpler cases; 9% conversion rate (2 laparotomy, 1 vaginal); mean Hb drop 2 g/dL; mean hospital stay 4 days
7.	Leon et al. [9]	126	77	49	Fellow console time, docking time, console time/docking time ratio, steps performed, number of control switches, complications	Single console: 4 (5.2%); Dual console: 5 (10.2%); no significant difference	Median console time: single 39 min vs. dual 54 min; docking time: single 84 min vs. dual 110 min; case time: single 142 min vs. dual 179 min (no significant adj. diff.)	MIGS fellows (PGY5‚Äì6)	Dual console increased fellow console time (+ 25.8 min), higher console/docking ratio, more steps performed (OR 3.37), more surgical takeovers (OR 3.53) without ‚Üë complications or case time; improved ‘hands-on’ training opportunities
8.	Margueritte et al. [8]	34 trainees/24 patients	0	34	Suturing times; simulation scores; questionnaire feedback; safety outcomes	2 intraoperative incidents: 1 hemorrhage (500 ml blood loss), 1 bladder wound; No conversions to laparotomy; No re-operations within 30 days	Mean suturing time 1st procedure: 3 min 38s; 2nd procedure: 3 min 14s (significant improvement *p* = 0.033); Total operating time: 165.8 ± 34.9 min; Console time: 135.8 ± 34.9 min	OB-GYN residents (3rd-5th year) and fellows with minimal robotic experience (90% had < 10 prior robotic cases)	100% found dual console helpful; 96.8% would recommend training; Simulation scores: Camera targeting 65.5%, Ring and rail 76.9%, Suture sponge 54.8%; Training included 3 half-days: simulation + 2 in vivo procedures
9.	Morgan et al. [20]	381 patients	185	196	Operative time; intraoperative complications; postoperative complications; estimated blood loss; continence; erectile function; biochemical recurrence; surgical margins	Intraoperative: Single 8.6% vs. Dual 1.5% (*p* < 0.0001); Postoperative: Single 14.1% vs. Dual 6.6% (*p* = 0.03); Clavien ≥3a: Single 7.0% vs. Dual 1.0% (*p* = 0.003); No conversions to open surgery	Single console: 222 ± 43 min vs. Dual console: 171 ± 44 min (*p* < 0.0001); Mean decrease of 51 min with dual console	5th year urology residents (chief residents) performing > 50% of each case after attending surgeon’s learning curve established (> 250 cases)	Dual console remained significant predictor of reduced operative time and intraoperative complications on multivariate analysis; No differences in oncologic outcomes (positive margins, biochemical recurrence) or functional outcomes (continence, erectile function) between groups; Composite complications: Single 17.3% vs. Dual 7.7% (*p* = 0.005)
10.	Williams et al. [11]	126 patients	0	126	Console time; intraoperative adverse events; estimated blood loss; blood transfusion; hospital stay; catheter duration; Clavien-Dindo complications; surgical margins; PSA detectability	Overall complication rate 23% (CD1: 9.5%, CD2: 8.7%, CD3b: 3.2%, CD4a: 1.6%); Intraoperative adverse events: 2 cases (1 caecal injury, 1 blood loss 1.5 L); No significant difference between trainee-led vs. specialist-led cases	Console time: Trainee-led 172.4 ± 57.9 min vs. Specialist-led 152.3 ± 60.9 min (*p* = 0.1489); No significant difference between groups	Fellowship level or penultimate year urology trainees vs. specialist urologists; Trainee-led cases defined as > 75% of 13 operative steps completed by trainee (39 cases, 31%)	Modular training approach with 13-step progression; Positive surgical margins: 26.2% overall (T2: 11.3%, T3: 37.5%); PSA undetectable at 6 weeks: 94.4%, at 12 weeks: 94.4%; No conversions to open surgery; Results comparable to international benchmarks despite trainee involvement
11.	Takahashi et al. [14]	16 surgeons (8 proctors + 8 operators)	0	16 surgeons	Operation times (proctor vs. operator) Dual cockpit switching times 5-point subjective rating scale for delay tolerance Modified System Usability Scale (mSUS) scores Robot Usability Scores Communication delay tolerance (0ms, 50ms, 100ms, 150ms, 200ms)	Not applicable (animal study with no clinical complications reported)	Proctor operation times: 216s (0ms) to 302s (150ms) delay - no significant difference (*p* = 0.247) Operator operation times: 433s (0ms) to 613s (200ms) delay - no significant difference (*p* = 0.608) Switching times: 2.82s to 4.19s across all delay conditions - no significant difference (*p* = 0.248)	Proctors: 8 specialists certified by Japan Robotic Surgery Society Operators: 8 trainee surgeons with no prior robotic surgery experience	Proctors rated delays ≥ 150ms as difficult for instruction (5-point scale: 3.8 at 150ms, 3.0 at 200ms, *p* < 0.05) Operators tolerated delays better (4.8 at 150ms, 4.3 at 200ms, *p* = 0.017) No significant difference in mSUS scores between proctors and operators (37.8 vs. 37.8, *p* = 0.779) Robot Usability Scores similar between groups (29.9 vs. 34.4, *p* = 0.261) Telesurgery with dual console deemed practical for remote surgical guidance Maximum acceptable delay for instructors: <150ms; operators could receive guidance even at 200ms delay
12.	Smith et al. [21]	222	106 (laparoscopic)	116 (robotic dual-console)	Total surgical time; total OR time; EBL; lymph nodes retrieved; length of stay; complications	Intraoperative: 6% vs. 2.5% (*p* = 0.201); Postoperative: 12% vs. 19% (*p* = 0.199); Conversion to open: 4.7% vs. 2.5%	Total surgical time: 131 vs. 110 min (*p* < 0.0001) favoring robotic; Total OR time: 172 vs. 175 min (*p* = 0.6)	Gynecologic oncology fellows (first and second year clinical fellows) working with attending physicians	Robotic surgery had significantly lower EBL (157 vs. 94 ml, *p* < 0.0001). Dual-console allowed fellow education without compromising outcomes. Similar lymph node yields and length of stay

### Skill acquisition and learning curve

It was observed that three studies documented accelerated skill acquisition through dual console training systems [12–14]. Jackson et al. reported that trainees achieved proficiency in advanced surgical tasks, such as hiatal dissection, within approximately 5 cases when using dual console systems [12].

The qualitative analysis by Cristofari et al. identified key elements contributing to skill development, including enhanced trainee communication, improved three-hand fluency, and increased situational awareness facilitated by dual console interaction [13].

Takahashi et al. demonstrated that dual console systems remained effective for surgical instruction even under simulated telesurgery conditions with communication delays up to 150 milliseconds, suggesting robust educational utility across various training environments [14].

### Operative efficiency and exposure

Overall, four studies reported outcomes in relation to operative efficiency (33.3%).The impact of dual console training on operative efficiency showed mixed results across studies.

Morgan et al. reported a significant 51-minute reduction in operative time when chief urology residents used dual console systems compared to single console training (171 ± 44 min vs. 222 ± 43 min, *p* < 0.0001) [20].

Conversely, Leon et al. outlined that while dual console training increased fellow console time by 25.8 min and total case time, the differences were not statistically significant after adjustment for case complexity [9].

Smith et al. demonstrated that robotic procedures with dual console fellowship training resulted in significantly shorter surgical times compared to laparoscopic approaches (110 vs. 131 min, *p* < 0.0001), while maintaining similar total operating room times [21].

Landry et al. observed a non-significant trend toward reduced operative times in dual console colorectal cases (200.6 ± 61.5 min vs. 220.0 ± 74.5 min, *p* = 0.111) [22]. The variation in operative time outcomes likely reflects differences in surgical complexity, trainee experience levels, and the degree of trainee independence across different studies and specialties.

#### Patient safety and complication rates

Overall, six studies reported outcomes in relation to complication rates (50%). Complication rates varied across studies but generally demonstrated acceptable safety profiles for dual console training.

Morgan et al. reported significantly lower intraoperative complications with dual console training compared to single console (1.5% vs. 8.6%, *p* < 0.0001) and reduced postoperative complications (6.6% vs. 14.1%, *p* = 0.03). Major complications (Clavien ≥3a) were also significantly lower in the dual console group (1.0% vs. 7.0%, *p* = 0.003) [20].

Williams et al. reported an overall complication rate of 23% in their dual console prostatectomy series, with only 2 intraoperative adverse events among 126 patients [11].

In gynecologic surgery, complication rates were generally low across dual console studies. Klapczynski et al. reported no conversions to laparotomy and no intra- or postoperative complications in their series of 7 resident-performed robotic hysterectomies [7].

Margueritte et al. documented 2 intraoperative incidents (hemorrhage and bladder injury) among 34 trainees but no conversions or reoperations within 30 days [8]. Leon et al. demonstrated a non-significant difference in complication rates between single and dual console groups in MIGS fellowship training (5.2% vs. 10.2%) [9].

### Educational effectiveness

Overall, three studies reported outcomes in relation to training quality (25%). Trainee and instructor satisfaction with dual console training was universally positive across studies that assessed these outcomes. Margueritte et al. reported that 100% of trainees found dual console training helpful, with 96.8% recommending the training approach to others [8]. Jackson et al. noted higher trainee satisfaction due to maximized console time, eliminating the traditional bedside-only phase of robotic surgery learning. The study also highlighted increased team interactivity and parallel learning opportunities for both faculty and trainees [12].

Cristofari et al. identified both advantages and challenges in dual console training through qualitative analysis. While the system facilitated enhanced communication and learning opportunities, it also created tension between maintaining a quiet, efficient operating room environment and the need for verbalization during training. The study suggested incorporating video-based self-confrontation debriefing to enhance reflection and skill acquisition [13].

### Implementation strategies

Five studies described specific strategies for integrating dual-console systems into surgical training curricula [7–9, 11, 12]. The most commonly reported approach was a modular, stepwise framework in which trainees initially observed from the second console before progressively assuming control of defined surgical steps under direct instructor supervision. Margueritte et al. implemented a structured curriculum comprising simulation exercises followed by in-vivo dual-console steps, with trainees performing increasingly complex components over successive cases [8]. Klapczynski et al. described a graduated autonomy model where residents performed resident-led robotic hysterectomy with real-time instructor oversight and the capacity for immediate takeover [7]. Jackson et al. outlined a dual-console paradigm for HPB surgery in which trainees bypassed the traditional bedside-only phase, gaining console access from the outset and progressing through defined tasks within approximately five cases [12]. Williams et al. formalised this into a 13-step modular mentorship programme for robot-assisted laparoscopic prostatectomy, with clear competency thresholds at each module [11]. Leon et al. documented that structured dual-console training led to significantly higher fellow console time and more surgical steps performed per case compared to single-console programmes, suggesting that systematic integration of dual-console systems produces measurable gains in operative exposure [9].

### Implementation barriers

Four studies documented barriers to the adoption of dual-console training systems [5, 11, 12, 22]. The most frequently cited obstacle was financial cost: dual-console systems require acquisition of a second surgeon console, representing a substantial capital investment that many institutions, particularly those in resource-limited or low-volume settings are unable to justify based on current evidence alone. Equipment availability was a related concern, with some programmes reporting insufficient dual-console access to ensure equitable case allocation across trainees. Curricular integration presented a further challenge, as existing robotic surgery training pathways were generally designed around single-console proctoring models and required restructuring to accommodate dual-console workflows, including modifications to case scheduling, faculty roles, and trainee assessment processes. Faculty development was also identified as a key barrier: instructors require specific training in dual-console teaching techniques, including strategies for verbal guidance, takeover decisions, and real-time assessment – skills distinct from conventional surgical supervision. Finally, Landry et al. noted that despite potential educational benefits in colorectal surgery, routine uptake was limited by logistical constraints within busy operating room environments [22].

### Specialty-specific outcomes

Overall, all studies reported outcomes in relation to specialty specific outcomes (100%). Gynecologic surgery studies demonstrated consistent benefits of dual console training across various procedures. Leon et al. showed increased console time, higher console-to-docking ratios, and more surgical steps performed by MIGS fellows without compromising outcomes [8]. Smith et al. found that dual console robotic training in gynecologic oncology resulted in lower estimated blood loss compared to laparoscopic training (94 vs. 157 ml, *p* < 0.0001) while maintaining similar lymph node retrieval rates and length of stay [21].

In urologic surgery, dual console training showed particular promise for complex procedures. Morgan et al. demonstrated maintained oncologic outcomes with dual console training, including similar positive surgical margin rates and biochemical recurrence rates [20]. Williams et al. achieved positive surgical margin rates (26.2%) and PSA undetectability rates (94.4%) comparable to international benchmarks despite significant trainee involvement in procedures [11].

The experimental study by Takahashi et al. explored the feasibility of dual console training under telesurgery conditions, finding that communication delays up to 150 milliseconds were acceptable for instructors, while trainees could receive effective guidance even at 200 milliseconds delay [14]. This suggests potential applications for remote surgical education and mentorship programs.

### Risk of bias assessments

Risk of bias assessments are outlined in *Supplementary Material 1* while publication bias assessments are described in *Supplementary Material 2*.

## Discussion

Across a range of surgical specialties and training levels, this systematic review summarises the most recent data on the installation, results, clinical outcomes and educational impact of dual-console robotic surgical systems. Together, the results point to dual-console training as a promising adjunct to improve surgical trainees’ exposure to operations, technical skill acquisition, and educational satisfaction without negatively impacting procedural efficiency or patient safety [1–4].

### Training outcomes

These data suggest that dual-console systems offer a distinctive learning environment that promotes interactive, real-time learning while upholding a high standard of procedural safety. Trainees reported quantifiable gains in technical performance across several investigations, such as higher autonomy scores, faster suturing times, and enhanced involvement in operative phases [5, 7–10]. According to these results, the dual-console system facilitates trainees to participate in complicated surgical tasks earlier and more meaningfully while also promoting the development of essential robotic competencies. Notably, stepwise autonomy and knot-tying times illustrated significant gains in the reports by both Klapczynski et al. [7] and Margueritte et al. [8], indicating gradual technical development during brief training sessions. Crusco et al.’s simulation-based RCT [10] also demonstrated the qualitative benefit of “virtual hands” guidance, highlighting the usefulness of dual-console systems in offering structured and interactive mentorship that transcends time-based metrics, even though it did not reveal significant differences in raw task times. These findings align with broader, recent syntheses of robotic training, which report that structured curricula combining simulation, stepwise clinical exposure, and objective assessment for example the Global Evaluative Assessment of Robotic Skills or Objective Structured Assessment of Technical Skill (GEARS/OSATS) consistently improve skill acquisition compared with ad-hoc training approaches [26, 27].

The influence of dual-console training on operative efficiency is context-dependent. For example, Morgan et al. reported significantly reduced operative times with dual-console use [20], whereas Landry et al. [22] and Leon et al. [9] observed non-significant increases in operative duration, largely attributable to greater trainee participation. These findings suggest that efficiency outcomes vary according to case complexity and trainee involvement. While some studies (e.g., Landry et al. [22], Leon et al. [4]) reported a non-significant trends towards longer operative durations, which likely reflect increased trainee participation and learning opportunities, other studies (e.g., Morgan et al. [20]) proceed to demonstrate that this training significantly reduced operative times and complication rates with dual-console systems, indication the long-term benefit if such platforms. The safety of dual-console training in both routine and complex procedures was further supported by the data suggesting that increases in operating time did not result in higher complication rates or unfavorable patient outcomes. The idea that dual-console training may be incorporated into high-acuity surgical settings without adversely affecting clinical outcomes— including oncologic metrics, such as positive margin rates and recurrence—is further supported by Williams et al. [11] and Smith et al. [1, 2]. Furthermore, recent registry reports demonstrate that integrating dual-console training modules provide greater trainee console exposure without increased adverse events [28, 31], thus ratifying the results of this study.

Maintaining or even enhancing patient safety through dual-console training is a recurring theme in the literature. Morgan et al. [20] demonstrated significantly lower intraoperative and postoperative complication rates with dual-console systems, while Williams et al. [11] reported safe outcomes in robotic prostatectomy despite substantial trainee involvement. Comparable findings were reported by Bolger et al. [34], whose evaluation of dual-console robotic platforms similarly demonstrated safe clinical outcomes while supporting increased trainee engagement. The large, retrospective cohort study by Morgan et al. [20] demonstrated significantly lower intraoperative and postoperative complication rates with dual-console training, which has also been ratified and supported in data presented by several smaller studies. These findings suggest that dual-console systems facilitate prompt instructor intervention during critical operative phases, reducing the risk of technical errors. Contemporary consensus and curricular guidance also emphasise that trainee autonomy should be graduated and competency-based, with dual-console supervision recommended as a means to allow earlier hands-on participation while preserving safety [29, 33].

### Educational effectiveness

The overwhelmingly positive feedback from instructors and trainees suggests that dual-console systems substantially improve the quality of surgical education. Reports of increased console time, reduced frustration, and better team dynamics indicate a more engaged and confident learner [8, 12]. Dual-console integration also facilitates a faster learning curve, as evidenced by Jackson et al. [12], where trainees bypassed the conventional “bedside-only” phase of robotic training.

Recent survey and mixed-method studies confirm these impressions, reporting that trainees exposed to structured dual-console curricula express higher confidence and satisfaction compared with those relying mainly on bedside assistance or simulation [30–32].

### Implementation strategies

The studies included in this review collectively demonstrate that dual-console training can be successfully integrated across diverse surgical specialties and training levels when implemented through structured, stepwise frameworks. Programmes that combined simulation-based preparation with graduated in-vivo dual-console exposure, such as those described by Margueritte et al. [8] and Williams et al. [11], reported consistent improvements in trainee operative exposure and autonomy. Jackson et al.’s dual-console paradigm for HPB surgery notably allowed trainees to reach proficiency in advanced tasks within approximately five cases, suggesting that well-designed integration can dramatically accelerate the learning curve compared to traditional single-console supervision [12]. Critically, these implementation strategies maintained or improved patient safety outcomes, supporting the feasibility of systematic dual-console integration in high-acuity settings. Recent consensus documents further recommend modular competency frameworks and routine use of objective assessment tools as essential enablers of successful implementation [26, 29].

### Implementation barriers

Despite the demonstrated benefits, the adoption of dual-console systems remains variable, limited by logistical, financial, and curricular challenges [5, 11, 12]. The high cost of acquiring a second console requires institutions to carefully weigh investment against educational benefit. Furthermore, effective integration into curricula requires restructuring to ensure equitable case allocation, faculty engagement, and standardized assessment. Recent programmatic evaluations emphasise three critical enablers for successful dual-console implementation: (1) modular competency frameworks, (2) formal faculty development in teaching and takeover strategies, and (3) routine use of objective assessment tools [26, 29]. Pilot programs demonstrate that these strategies can make dual-console integration feasible even in resource-constrained settings [31]. Emerging evidence also suggests that dual-console platforms may support telesurgical mentorship, with feasibility demonstrated even under moderate communication delays [25]. This could extend training opportunities to geographically isolated centers, though medico-legal, technical, and assessment barriers remain [12, 22].

### Limitations

While our synthesis and recent reports are encouraging, several limitations must be acknowledged. The heterogeneity of study designs, outcome measures, and trainee experience complicates direct comparisons and precludes pooled analysis. Most available evidence is observational, single-center, and underpowered for patient-level outcomes, with only one RCT previously performed in the academic literature [10]. Publication and selection bias remain possible, given the predominance of single-arm case series and the frequent reporting of favorable educational outcomes [7, 8]. Recent systematic reviews of robotic training similarly highlight the need for standardized outcome tools, preregistered protocols, and economic analyses to strengthen the evidence base [26, 27].

### Future directions

Future translational research efforts should prioritize multicenter RCT, standardized assessment metrics (e.g., GEARS, OSATS), and longitudinal follow-up to evaluate long-term skill retention and independent operative performance. Long term cost-effectiveness analyses comparing dual-console training with traditional or simulation-only models are needed to guide institutional investment [26, 27]. Furthermore, customized curricula and faculty development programs will be essential as robotic surgery expands across specialties. Integration of video review, telesupervision, and Artificial Intelligence (AI) driven feedback may further enhance the scalability and effectiveness of dual-console training [12, 29].

In conclusion, dual-console robotic systems represent a significant advancement in surgical education, offering a safe and structured environment for hands-on trainee engagement while maintaining patient outcomes. Evidence across surgical specialties indicates improvements in skill acquisition, operative exposure, and trainee satisfaction, with possible reductions in intraoperative errors and complications [1–4, 8, 10–12]. Although operative times vary, this largely reflects greater trainee involvement rather than inefficiency. Despite clear educational benefits, widespread adoption remains constrained by financial, logistical, and curricular barriers [5, 11]. The contemporary evidence base, which is dominated by small observational studies, highlights the urgent need for standardized, multicenter studies with long-term follow-up to fully establish the role of dual-console robotic training platforms [10, 26, 27].

## Supplementary Information

Below is the link to the electronic supplementary material.

Supplementary Material 1

## Data Availability

No datasets were generated or analysed during the current study.
