# Supplementary material for: Overview of dual console robotic training for surgical residents: a systematic review
Source: J Robot Surg. 2026 Apr 10;20(1):429. doi: 10.1007/s11701-026-03331-7 (PMC13068738; doi:10.1007/s11701-026-03331-7)
Supplement: Supplementary file 1 — Supplementary Material 1 [file 11701_2026_3331_MOESM1_ESM.docx]

Consolidated Risk of Bias Report — Dual Console Robotic Surgery Studies

Risk-of-bias assessment tools were applied according to study design. The Newcastle–Ottawa Scale (NOS) was used only for observational studies. ROB 2.0 was used only for randomized controlled trials (RCTs). Bias was not assessed for expert consensus/opinion pieces or preclinical/experimental (non-clinical) studies, as these fall outside the scope of standard clinical RoB tools. Each included study was assessed with one appropriate primary tool only.

# Summary Table (Newcastle–Ottawa Scale)

| Study | Design | NOS Sel (0–4) | NOS Comp (0–2) | NOS Out (0–3) | NOS Total (0–9) | Overall Judgment |
| --- | --- | --- | --- | --- | --- | --- |
| Morgan et al. (2015) | Retrospective before-after cohort (single surgeon, N=381) | 4 | 1 | 3 | 8 | NOS 8/9. ROBINS-I: Moderate risk (confounding by time/selection). |
| Leon et al. (2022) | Prospective non-randomized comparative cohort | 4 | 1 | 3 | 8 | Low risk (prospective collection, controlled analyses) |
| Landry et al. (2022) | Retrospective cohort (single surgeon, colorectal) | 4 | 0 | 3 | 7 | Some concerns (retrospective, limited adjustment) |
| Klapczynski et al. (2021) | Prospective single-arm case series (dual-console hysterectomy; n=7) | 2 | 0 | 2 | 4 | High risk (very small sample, no control) |
| Marengo et al. (2012) | Prospective cohort / case series (double-console, n=33) | 3 | 0 | 2 | 5 | Moderate-to-high risk (single-arm, no comparator) |
| Smith et al. (2012/2013) | Retrospective single-arm case series (first 50 dual-console hysterectomies). | 2 | 0 | 2 | 4 | High |
| Williams (2025) | Prospective observational cohort comparing trainee (dual-console supervised) vs specialist (independent) operators. | 4 | 1 | 3 | 8 | Moderate |
| Margueritte (2019/2020) | Prospective case series (curriculum + in-vivo dual-console steps; feasibility & survey endpoints). | 3 | 0 | 2 | 5 | Moderate |

# Study-by-Study Notes

## Crusco et al. (2014)

Design: Randomized parallel-group trial (novice training)

Primary Tool: ROB-2 (randomized trial)

Crusco et al. (2014), the only randomized controlled trial included in this review, was assessed using the ROB 2.0 tool and judged to have some concerns for overall risk of bias. Randomization was conducted using a computer-generated sequence by a statistician, and baseline demographic and experiential characteristics were well balanced between the single- and dual-console groups, indicating a low risk of bias arising from the randomization process. The intervention was delivered as intended, with no evidence of deviations from assigned groups, and outcome data were complete for all randomized participants. The primary outcome (task completion time) and secondary outcomes were objectively measured and are unlikely to have been influenced by lack of blinding. However, as no trial registration or prespecified protocol was reported, selective reporting of outcomes cannot be fully excluded, which accounts for the overall judgment of some concerns.

## Morgan et al. (2015)

Design: Retrospective before-after cohort (single surgeon, N=381)

NOS: Selection 4, Comparability 1, Outcome 3 (Total 8/9)

Overall Judgment: NOS 8/9

## Leon et al. (2022)

Design: Prospective non-randomized comparative cohort

NOS: Selection 4, Comparability 1, Outcome 3 (Total 8/9)

Primary Tool: NOS (cohort)

Overall Judgment: Low risk (prospective collection, controlled analyses)

## Landry et al. (2022)

Design: Retrospective cohort (single surgeon, colorectal)

NOS: Selection 4, Comparability 0, Outcome 3 (Total 7/9)

Primary Tool: NOS (cohort)

Overall Judgment: Some concerns (retrospective, limited adjustment)

## Klapczynski et al. (2021)

Design: Prospective single-arm case series (dual-console hysterectomy; n=7)

NOS: Selection 2, Comparability 0, Outcome 2 (Total 4/9)

Overall Judgment: High risk (very small sample, no control)

## Marengo et al. (2012)

Design: Prospective cohort / case series (double-console, n=33)

NOS: Selection 3, Comparability 0, Outcome 2 (Total 5/9)

Overall Judgment: Moderate-to-high risk (single-arm, no comparator)

### Smith et al. (2012/2013)

NOS was utilized Overall: High RoB (single-arm; learning-curve/selection bias).

### Jackson (2020)

Expert opinion / educational commentary

No risk-of-bias assessment performed

### Williams (2025)

NOS total 8/9. Overall: Moderate RoB (residual confounding by operator/case-mix).

Rationale: Comparative observational design but non-randomized; residual confounding plausible. Downgraded to Moderate ROB despite good prospective methods.

### Margueritte (2019/2020)

Overall: Moderate RoB (no comparator; mixed clinical and perception outcomes).

Rationale: Feasibility/prospective series without comparator; outcomes include trainee perceptions.

### Cristofari (2021/2022)

Qualitative video-based communication analysis

No clinical RoB assessment performed (qualitative rigor described narratively)

### Takahashi (2024)

Preclinical experimental/simulation study (animal model)

No clinical RoB assessment performed

# Traffic-Light Grid (Domains & Overall)

| **Study** | **Selection** | **Comparability** | **Outcome** | **Overall** |
| --- | --- | --- | --- | --- |
| Crusco et al. (2014) | 🟢 Low | 🟢 Low | 🟡 Some concerns | 🟡 Some concerns |
| Morgan et al. (2015) | 🟢 Low | 🟡 Some concerns | 🟢 Low | 🟡 Some concerns |
| Leon et al. (2022) | 🟢 Low | 🟡 Some concerns | 🟢 Low | 🟢 Low |
| Landry et al. (2022) | 🟢 Low | 🔴 High | 🟢 Low | 🟡 Some concerns |
| Klapczynski et al. (2021) | 🟡 Some concerns | 🔴 High | 🟡 Some concerns | 🔴 High |
| Marengo et al. (2012) | 🟡 Some concerns | 🔴 High | 🟡 Some concerns | 🔴 High |
| Smith et al. (2012/2013) | 🟡 Some concerns | 🔴 High (N/A: no comparator) | 🟡 Some concerns | 🔴 High |
| Williams (2025) | 🟢 Low | 🟡 Some concerns | 🟢 Low | 🟡 Moderate |
| Margueritte (2019/2020) | 🟡 Some concerns | N/A | 🟡 Some concerns | 🟡 Moderate |
|  |  |  |  |  |
